# The Consequences of Being in an Infectious Biofilm: Microenvironmental Conditions Governing Antibiotic Tolerance

**DOI:** 10.3390/ijms18122688

**Published:** 2017-12-12

**Authors:** Majken Sønderholm, Thomas Bjarnsholt, Maria Alhede, Mette Kolpen, Peter Ø. Jensen, Michael Kühl, Kasper N. Kragh

**Affiliations:** 1 Costerton Biofilm Centre, Department of Immunology and Microbiology, University of Copenhagen, DK-2200 Copenhagen, Denmark; ms@sund.ku.dk (M.S.); Tbjarnsholt@sund.ku.dk (T.B.); MVG@sund.ku.dk (M.A.); Mette.Kolpen@regionh.dk (M.K.); Peter.Oestrup.Jensen@regionh.dk (P.Ø.J.); 2 Department of Clinical Microbiology, Copenhagen University Hospital, DK-2100 Copenhagen, Denmark; 3 Marine Biology Section, Department of Biology, University of Copenhagen, DK-3000 Elsinore, Denmark; mkuhl@bio.ku.dk; 4 Climate Change Cluster, University of Technology Sydney, Ultimo NSW 2007, Australia

**Keywords:** biofilm, microenvironmental, antibiotic tolerance

## Abstract

The main driver behind biofilm research is the desire to understand the mechanisms governing the antibiotic tolerance of biofilm-growing bacteria found in chronic bacterial infections. Rather than genetic traits, several physical and chemical traits of the biofilm have been shown to be attributable to antibiotic tolerance. During infection, bacteria in biofilms exhibit slow growth and a low metabolic state due to O_2_ limitation imposed by intense O_2_ consumption of polymorphonuclear leukocytes or metabolically active bacteria in the biofilm periphery. Due to variable O_2_ availability throughout the infection, pathogen growth can involve aerobic, microaerobic and anaerobic metabolism. This has serious implications for the antibiotic treatment of infections (e.g., in chronic wounds or in the chronic lung infection of cystic fibrosis patients), as antibiotics are usually optimized for aerobic, fast-growing bacteria. This review summarizes knowledge about the links between the microenvironment of biofilms in chronic infections and their tolerance against antibiotics.

## 1. Introduction

**Figure 1:**
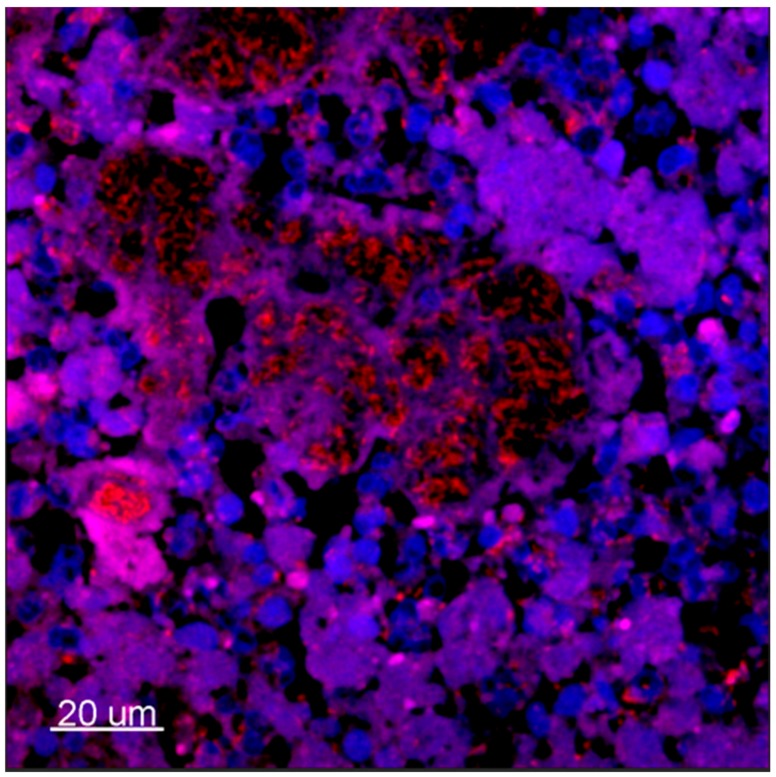
Confocal laser-scanning microscopy micrography of ex vivo lung tissue from a *P. aeruginosa*-infected CF patient. Tissue was stained with peptide nucleic acid fluorescence in situ hybridization (PNA-FISH) probes specific for *P. aeruginosa* with a red Texas-Red flourophor and counterstained with blue (4′,6-diamidino-2-phenylindole) DAPI for eukaryotic nucleus. 630×. [19].

In medical microbiology, the clinical implications of bacterial biofilms are particularly pronounced in chronic infections [1]. Biofilms in chronic infections can be either surface associated or suspended [2]. Surface-associated biofilms are found in a number of cases, such as on teeth [3] or in implant-associated infections [4,5,6] where bacteria reside on the surface of, for example, an indwelling catheter [7,8]. Such surface colonization of pathogens forms large biofilms of up to 1200 µm in thickness [9]. In contrast, chronic lung infection in cystic fibrosis (CF) patients [9,10], chronic wounds [9], soft tissue filler-related infections [11,12], and chronic middle-ear infections [13,14] carry biofilms as non-attached, relatively small, suspended cell aggregates (~2–200 µm in diameter) [9,10,15,16,17]. Such suspended biofilms are typically embedded in a secondary matrix that consists of pus, wound-bed sludge or CF mucus, and the bacterial cell aggregates are often surrounded by inflammatory cells such as polymorphonuclear leukocytes (PMNs) [10,18,19] (Figure 1).

The advent of antibiotics following the discovery of penicillin by Alexander Fleming in 1929 was a major breakthrough in the treatment of bacterial infections, which were a major cause of morbidity and mortality in the general population. However, resistance to antibiotics was already reported in the 1940s [20] and has been increasing ever since. Resistance to antibiotics is caused by inherited or acquired genetic changes via mutations or genetic transfer between bacteria [21,22] such as that caused by efflux pumps, which are transport proteins involved in the extrusion of toxic substrates [23]. Antibiotic resistance continues to receive much attention, but it should not be confused with antibiotic tolerance. Whereas resistance is an irreversible change to the genome of the bacteria, tolerance is a reversible state of the bacteria that enables them to survive antibiotic treatment [24].

When antibiotic therapy fails [25,26,27] an infection can become chronic due to several factors inherent to biofilm-growing bacteria. Høiby et al. (2010) describes chronic bacterial infections, including foreign-body infections, as infections, (i) that persist despite antibiotic therapy and the innate and adaptive immune and inflammatory responses of the host, and (ii) that are characterized, in contrast to colonization, by an immune response and persisting pathology [28]. In comparison to acute bacterial infections, chronic infections are described by slow progression and low-grade pathogenesis. Chronic bacterial infections can develop if the main defensive system is impaired. Such impairments include the disruption of the anatomical barrier (for example, skin or mucosal membranes) or deficiencies in the innate immune response (i.e., macrophages or neutrophils) which allow bacteria that would otherwise be rapidly cleared by the host to establish a chronic infection [29]. The deficiencies can be either congenital (for example, cystic fibrosis (CF)), acquired (for example, diabetes), or due to the presence of foreign-body implants [9,30]. Why some bacterial infections manage to establish chronic infections, which are difficult if not impossible to resolve, remains largely unanswered. However, increasing evidence points towards biofilm-growing bacteria as common denominators behind chronic infections [2]. 

When examining the literature on the lifestyle of bacteria living biofilms, one aspect is often repeated: slow growth and low metabolic state [2,19,31,32,33,34]. Biofilm-embedded bacteria in slow-growing or even in dormant states in infections will have profound effect on the effectiveness of antibiotics [35,36,37]. Stratified growth patterns in infectious biofilms may point towards a scheme in which growth conditions have a key influence on the antibiotic tolerance exhibited by cells with low growth rates. This makes it paramount to shed light on the microenvironment surrounding bacterial biofilms in chronic infections.

## 2. The Microenvironment of Chronic Biofilm Infections

While different chronic bacterial infections throughout the human body may appear diverse, they share several similarities in terms of the characteristic growth pattern and microenvironment of the pathogenic bacteria. Many chronic bacterial infections thus exhibit the presence of small suspended biofilm aggregates surrounded by a high-density of inflammatory cells and imbedded in pus or tissue, which present an hypoxic or anoxic habitat [9,38]. Chronic lung infections in CF patients present a well-explored example of such conditions.

An adult at rest inhales about 11,000 liters of air every day (~0.5 liters air per breath, 15 breaths per minute); however, anoxic zones in the lungs of CF patients can still be found. A combination of oxygen (O_2_) consumption by epithelial cells [17]—with excessive O_2_ consumption by active PMNs engaged in the respiratory burst or production of nitric oxide (NO) directed against bacteria [39,40]—can lead to the development of hypoxic and anoxic conditions in CF lungs [17]. Furthermore, ex vivo samples of lungs from CF patients have revealed that the number of PMNs surrounding bacterial biofilms correlates negatively with the growth rate of *P. aeruginosa* [19]. As PMNs consume O_2_, these biofilms may experience strong O_2_ limitation, thereby rendering them less susceptible to antibiotic treatment. Reduced growth rates under hypoxia or anoxia in biofilms associated with chronic infections can have serious implications, as antibacterial treatment strategies are usually developed for aerobic bacteria [26]. The ability of the PMN response to impose slow bacterial growth is further supported by recent findings, showing that *Stenotrophomonas maltophilia*, *Staphylococcus aureus*, *Achromobacter xylosoxidans* and *Streptococcus pneumonia* also grow very slowly in the CF sputum [41,42].

**Figure 2:**
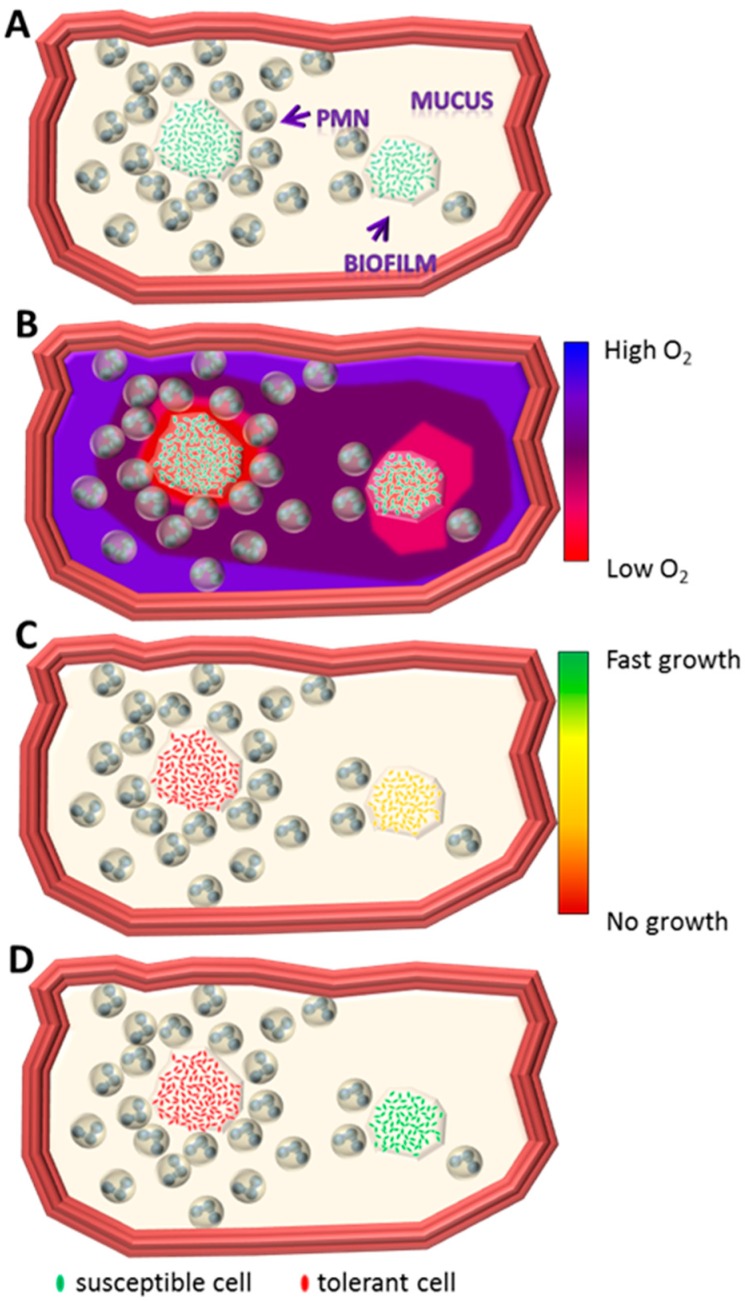
Schematic drawing of a biofilm-infected bronchial lumen based on ex vivo observation (Figure 1). (**A**) The bronchial lumen with two non-attached biofilms surrounded by polymorph nuclear leukocyte (PMN) infiltrated mucus; (**B**) oxygen gradient through the mucus towards the biofilm. High concentration of PMNs results in steeper oxygen gradients due to high consumption; (**C**) high concentration of PMNs around a biofilm results in slow-growing bacteria within the biofilm. Lesser concentration results in increased growth due to improved accessibility to oxygen; (**D**) differences in growth rate may result in differentiated tolerance towards antibiotics. Jensen et al. [46].

Few O_2_ measurements have been performed in situ in the CF lung [17] and in ex vivo sputum samples [40,43], and such measurements have revealed steep O_2_ gradients in the mucus constituting a secondary matrix of bacterial aggregates. This is further supported by in vivo observations of the upregulation of the global, O_2_-sensing, anaerobic regulator *Anr*-controlled genes of *P. aeruginosa* in CF sputum [38,44,45]. Although it is possible to measure O_2_ gradients in the first two to three branches of the bronchia, many low-O_2_ niches may exist on an even smaller scale than is currently measurable in vivo. Thus, an appropriate description of the microenvironment encountered by bacteria in chronic infections requires further analysis, as relatively small niches may represent large physical and chemical differences for the inhabiting bacteria (Figure 2). Altogether, the bacteria inhabiting chronic infections are faced with very variable environments that favor versatile metabolism and physiology and may strongly influence the growth and in turn the effectiveness of antibiotics. Kopf et al. show a very slow (median of 2.1 days per cells division) but extremely diverse growth rate even at the single-cell level for *S. aureus* in freshly harvested expectorates from a patient with CF [42]. At present, in vivo observations suggest that the availability of O_2_ or alternative electron acceptors is a core limiting factor for bacterial biofilm growth in chronic infections [19]. It is therefore vital that researchers and clinicians understand how bacterial metabolism can adjust to the accessibility of electron acceptors from fully oxic to anoxic conditions if we are to comprehend the development of antibiotic tolerance in chronic biofilm infections.

### 2.1. Aerobic Respiration

Aerobic respiration is the most energy-efficient form of respiration [47,48]: approximately 50% of the potential energy in the electron donor is conserved in the form of an ATP-generating proton-motive force [49]. The machinery for aerobic respiration consists of NADH dehydrogenase (Complex I), the quinone pool (UQ), the *bc*_1_ complex (Complex III), and cytochrome *c* (Cyt. *c*). Aerobic respiration requires terminal oxidases (Complex IV). For example, *P. aeruginosa* has five terminal oxidases that catalyze the four-electron reduction of molecular O_2_ to water; they differ in their affinity for O_2_, the efficiency of energy coupling, and the tolerance to various stresses where the high-affinity terminal oxidases are used under hypoxic conditions [49]. The *bo*_3_ oxidase (*cyo*) and the cyanide-insensitive oxidase (CIO) (*cio*) are quinol oxidases, whereas *cbb*_3_-1 (*cco1*), *cbb*_3_-2 (*cco2*) and *aa*_3_ (*cox*) are cytochrome c oxidases [48,50]. 

### 2.2. Anaerobic Respiration

**Figure 3:**
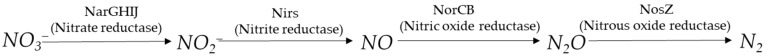
Denitrification enzymes that reduce NO_3_^−^ to N_2_ (nitrogen gas).

While O_2_ is the most energy-efficient electron acceptor, *P. aeruginosa* and many other bacteria can use other electron acceptors for respiration. Among these, nitrate (NO_3_^−^) respiration has an energy yield approaching that of O_2_ [49]. Anaerobic respiration by denitrification, where NO_3_^−^ is reduced to gases [51], is able to conserve only ~30% [49] of the potential energy of an electron donor for ATP-generation due to a possible loss of energy during the denitrification pathway. NO_3_^−^ is common in the environment and available in vivo [40,52,53] at physiological levels supporting growth [54]. PMNs are abundant in CF lungs and are reported to contribute NO_3_^−^ or nitrogen dioxide (NO_2_^−^) [55]. Reduction of NO_3_^−^ to NO_2_^−^ is a prerequisite to actual denitrification, where NOx are reduced to gases [51]. Denitrification relies on the same respiratory framework as aerobic respiration. All denitrifiers can respire aerobically, but specific enzymes are needed for denitrification [49]. *P. aeruginosa* has a complete set of denitrification enzymes that reduce NO_3_^−^ to N_2_ (nitrogen gas) [51], and show in Figure 3:

The primary CF pathogen, *P. aeruginosa*, can survive during O_2_ limitation by performing anaerobic respiration with NO_3_^−^. The production of the reactive oxygen species (ROS) superoxide (O_2_^−^) by PMNs—combined with endogenously produced nitric oxide by nitric-oxide synthases in lung tissue and PMNs—may result in the formation of peroxynitrite, which can decompose into NO_3_^−^ [56]. This is supported by observations of reduced exhaled NO from CF lungs [57]. Because of ROS production—and to a lesser extent because of the production of NO by the activated PMNs—hypoxic and anoxic zones are created in which aerobic respiration is limited due to the lack of O_2_ as an electron acceptor. However, the combination of ROS and NO production may alleviate the consequences associated with the limitation of aerobic respiration by the simultaneous production of NO_3_^−^, which may serve as an alternative electron acceptor, thereby allowing for ATP generation by anaerobic respiration [55]. The physiological concentration of NO_3_^−^ in CF lungs can reach up to 1 mM, as estimated from concentrations of NO_3_^−^ measured in freshly expectorated CF sputum [40,52,53]. The reported concentrations are based on bulk measurements. However, due to the highly varied spatio-temporal distribution of electron acceptors involved in respiration (e.g., O_2_ and N_2_O [40,43]), local NO_3_^−^ concentrations may be even higher. NO_3_^−^ concentrations as high as 100 mM have been shown to yield high bacterial densities in CF lungs [58,59]. However, physiologically relevant growth rates can be obtained by supplementing anoxic cultures of *P. aeruginosa* with far less than 1 mM NO_3_^−^ [54]. Similar to the CF environment, microsensor measurements of O_2_-concentration profiles in ex vivo chronic human wounds and in vivo chronic mouse wounds reveal steep O_2_ gradients [60]. Furthermore, anaerobic bacteria have been isolated from both expectorated CF mucus [61,62,63] and chronic wounds [64,65], thereby indicating the existence of hypoxic and anoxic zones. 

### 2.3. Substrate Level Phosphorylation

High levels of amino acids are present in CF sputum [66], and *P. aeruginosa* is able to utilize such amino acids as growth substrate in CF sputum [67]. *P. aeruginosa* can ferment arginine anaerobically such that arginine is degraded to ornithine, which generates 1 mol ATP from 1 mol arginine [68]. In comparison, the oxidation of 1 molecule of glucose can generate 36 molecules of ATP during aerobic respiration [69]. In contrast, fermentation of the Alfa-keto acid pyruvate does not supply *P. aeruginosa* with energy for growth, but it does enable the long-term survival of the bacterium [70,71], again adding to the remarkable persistence of *P. aeruginosa* in chronic infections. 

### 2.4. Microaerobic Metabolism

Microaerobic metabolism is not very well defined, but it may be described as an intermediate state in which the organism(s) simultaneously performs aerobic and anaerobic respiration or fermentation [72]. 

During hypoxic conditions, some microorganisms can perform so-called aerobic denitrification. Aerobic denitrification, or co-respiration of O_2_ and NO_3_^−^, was reviewed by Robertson and Kuenen and can be described as NO_3_^−^ respiration in the presence of O_2_ [73]. According to phylogenetic analysis, aerobic denitrifiers mainly belong to α-, β- and γ-*Proteobacteria* [74]. Alvarez-Ortega et al. [59] report that *P.* *aeruginosa* expresses overlapping gene sets depending on O_2_ concentration, and moreover that low O_2_ triggers denitrification genes. *P. aeruginosa* can thus switch flexibly between several types of electron acceptors [59]. This is consistent with *cbb_3_-1* being constitutively expressed [48], thereby enabling *P. aeruginosa* to adapt to variable levels of O_2_. Diverse chemical microniches may exist in close proximity within the same infection [19]. This complicates the treatment of chronic biofilm infections, as the bacteria do not exhibit a homogeneous physiological state but grow under a variety of conditions, probably resulting in varying growth rates.

## 3. Correlating the Microenvironment to Antibiotic Tolerance

One of the main targets for biofilm research has been the desire to understand the mechanisms behind tolerance towards antibiotics. The explanation for this enhanced tolerance is attributable to several physical and chemical traits of the biofilm, which enable bacteria to survive very high doses of antibiotics, usually exceeding the minimal inhibitory concentration (MIC) of the antibiotic many times. In fact, it has been reported that the MIC of biofilm-growing bacteria can be 100–1000 times higher than that of its planktonic counterpart [75,76,77]. Traditionally, MIC is determined by exposing bacteria to increasing concentrations of antibiotics and observing the level at which growth is inhibited [78,79]. However, MIC measured in vitro depends on growth conditions [24], thereby making it difficult to transfer such findings to in vivo settings. Furthermore, MIC is typically determined in a planktonic bacterial suspension, which does not represent the in vivo scenario in chronic infections, where biofilm-growing bacteria predominate [2]. However, in 1999, the first method for estimating the MBEC (minimal biofilm-eradication concentrations) growing on a surface was reported by Ceri et al. who used a Calgary Biofilm device (CBD) [80]. Since then, other methods, such as bioreactor-based models and flow cells systems, and definitions such as minimal biofilm inhibitory concentration have been reported, but a standardization of the procedures remains outstanding [27].

The matrix of the biofilm itself can be considered a physical barrier by conferring poor penetration of antibiotics, but several studies speculate that this is probably not a universal mechanism [32,81]. There is experimental evidence that negatively charged eDNA and several polysaccharide components of a biofilm matrix may contribute to shielding against positively charged aminoglycosides [82,83]. Additionally, studies have found indications of a genetic contribution to increased antimicrobial tolerance [84]. For example, consider efflux pumps [85], toxin-antitoxin modules [86], quorum sensing [87], modifying the charge of the cell surface [88], and active adaptation of expression as a response to nutrient limitation [89,90]. We note that genetic tolerance could play a role during biofilm growth, but we argue that within chronic infection in the physiological microenvironment may have a strong influence on the development of tolerance. 

From in vitro biofilm studies, we know that chemical heterogeneity can protect the biofilm from antibiotics [91,92]. In in vitro, surface-associated biofilms, thick *P. aeruginosa* biofilms harbor physiologically different subpopulations. Bacteria at the top of the biofilm are metabolically active—as determined by mRNA levels and selective green-fluorescence-protein (GFP) labelling of active bacterial cells—whereas biofilms at the bottom of the biofilm experience reduced growth rates and low mRNA levels [33]. Slow growth rates coincide with increased tolerance towards tobramycin and ciprofloxacin, whereas metabolically active bacterial cells are more susceptible [92]. The increased antibiotic tolerance of biofilms has been the focus of several studies, which have drawn various conclusions. Spoering et al. argue that the increased tolerance of biofilm bacteria towards antibiotics, as compared to planktonic bacteria, could be attributed to their reduced growth, and that biofilm bacteria and stationary-phase planktonic bacteria are equally tolerant to antibiotics [93]. On the other hand, Staudinger et al. [94] suggest that cell-aggregate formation, rather than the biofilm phenotype, is responsible for increased antibiotic and host-defense tolerance. Mikkelsen et al. [95] also question whether biofilms and planktonic bacterial cells are phenotypically different entities and observe that biofilm protein profiles resemble that of planktonic cells. They may simply represent different growth phases [95]. Furthermore, in *P. aeruginosa* biofilms, O_2_ limitation, and thus low metabolic activity, can be correlated with increased antibiotic tolerance [32,96]. Recently, Pabst et al. found that a similar pattern of local hypoxia and reduced growth rates affect the antibiotic tolerance of *S. aureus* [81]. 

The ability of *P*. *aeruginosa* to grow under O_2_ limitation, and its ability to utilize arginine and NO_3_^−^ during these conditions, for example, has several implications for antibiotic susceptibility. Tobramycin targets O_2_-dependent active transport, and its action should thus be inhibited during O_2_ limitation. However, in anoxic biofilm, the addition of an alternative electron acceptor such as NO_3_^−^ or arginine for fermentation [96,97] increases susceptibility to tobramycin, thereby indicating that the anti-bacterial activity of tobramycin also targets anaerobic metabolism to some degree. In fact, according to Borriello et al., O_2_ limitation could account for 62% of Ciprofloxacin tolerance, 69% of Tobramycin tolerance, 80% of Tetracycline tolerance and >110% in the case of Ceftazidime [96]. Furthermore, Connell et al. show that in vitro tolerance towards antibiotics already occurs in aggregates of just 100 bacteria, which is not enough to create anoxia inside the biofilm [98]. This indicates that O_2_ limitation is an important, but probably not the only, factor contributing to antibiotic tolerance in *P. aeruginosa*. Perhaps the low bacterial metabolism in anoxic regions contributes equally to the observed antibiotic tolerance of biofilms in vitro and in vivo. It has thus been suggested that stimulation of bacterial activity by supplementation of NO_3_^−^ or arginine could lead to increased susceptibility to tobramycin and ciprofloxacin when treating in vivo biofilms [97].

The fact that biofilm tolerance towards antibiotics is primarily attributable to a physico-chemical, rather than to a genetic trait is supported by studies showing that tolerance to antibiotics is reversible in vitro [11,99,100]. Antibiotic susceptibility can be restored if bacteria are released from biofilms [32,101] or if metabolism is activated by stimulating aerobic respiration, anaerobic respiration, or substrate-level phosphorylation in biofilm [97,102,103]. This aspect has been further demonstrated in a study, where transcriptional analysis has revealed a correlation between the genetic expression of planktonic growing bacteria and alginate-encapsulated, biofilm-growing bacteria supplemented with an alternative electron acceptor (NO_3_^−^) [44]. When the biofilm-growing bacteria were supplemented with NO_3_^−^, electron acceptor limitation was alleviated, thereby stimulating metabolism. When the alginate-encapsulated biofilm-growing bacteria were not supplemented with NO_3_^−^, the bacteria experienced electron-acceptor limitation, and the genetic-expression profile was very different from that of the planktonic-growing bacteria [44]. This suggests that it was the alleviation of electron-acceptor limitation—and thus stimulated metabolism rather than the biofilm-growing state of the bacteria—that affected the expression profiles. In fact, in vitro studies have shown that such alleviation of electron-acceptor limitation, which increases metabolism, in turn increases susceptibility towards the antibiotic tobramycin and ciprofloxacin [97,102,103]. This is in accordance with the fact that tobramycin and ciprofloxacin are antibiotics that target fast-growing bacteria [104,105]. In other words, the low metabolism of slow-growing bacteria antagonizes the effect of tobramycin.

## 4. Treatments to Tackle the Broad Spectrum of Growth Rate

Monotherapy with antibiotics (such as β-lactams and the aminoglycoside tobramycin) is effective only against active bacterial cells [76]. Due to the nature of chronic infections, treatment with single antibiotics is not effective, and several classes of antibiotics are often used in a combination to target bacteria in different metabolic states [2]. An example is the treatment of CF patients, where a combination of colistin and either ciprofloxacin or tobramycin is used. These combinations target different subpopulations of in vitro biofilms, as described below. While tobramycin and ciprofloxacin target bacteria in the outer active biofilm layer, colistin targets less active or dormant bacteria in the inner parts of biofilms [37,106]. 

**Figure 4:**
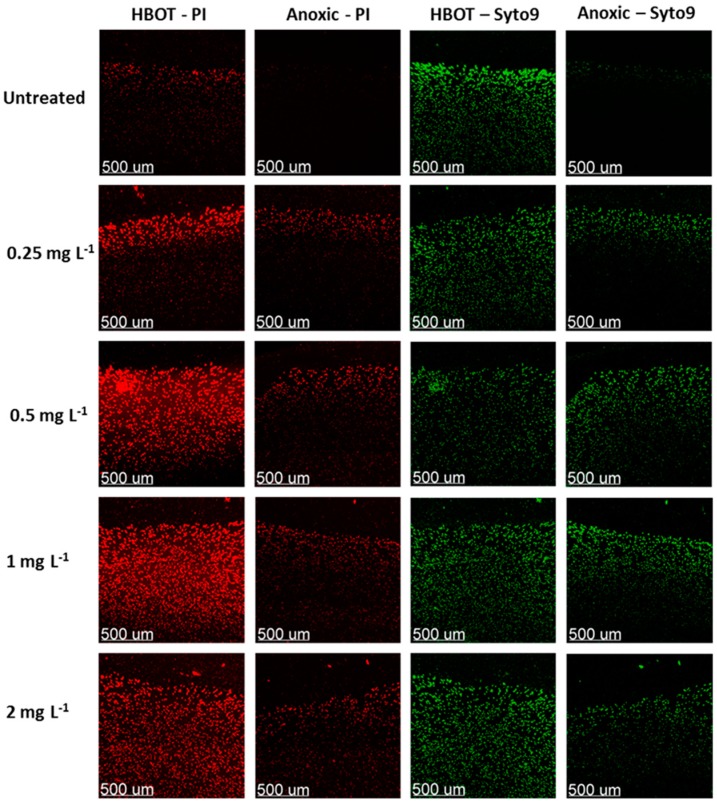
Lethality of a ciprofloxacin-treated *P. aeruginosa* biofilm during anoxic or Hyperbaric O_2_ treatment (HBOT) conditions. Dead or dying cells where stained red with Ppropidium iodide (PI) and live cells where stained green with SYTO9.

Another promising approach is to supply extra O_2_ to hypoxic environments [41,107,108] to overcome the antibiotic tolerance in established biofilms where dormant bacteria are present. Hyperbaric O_2_ treatment (HBOT) has previously been used as an adjuvant to ciprofloxacin on *P. aeruginosa* [109,110], but the underlying mechanism still needs to be investigated thoroughly. Application of HBOT has been shown to enhance the bactericidal activity of tobramycin to *S. aureus* in an endocarditis model [107], while ciprofloxacin has been shown to enhance the bactericidal activity in a *P. aeruginosa* biofilm model [41,108]. Kolpen et al. recently demonstrated that application of HBOT during ciprofloxacin treatment is accompanied by indications of restored aerobic bacterial respiration when hypoxia is established from hyperoxia after clinically relevant durations of HBOT [108]. This forced metabolic shift from anaerobic to aerobic respiration promotes the faster growth of *P. aeruginosa* under HBOT, as indicated by increased SYTO9 fluorescence intensity and bacterial aggregate size after HBOT (Figure 4). HBOT can thus be considered an adjuvant that enhances the activity of bactericidal antibiotics for several sites of biofilm infection (such as chronic lung infection, wounds, bones and biliary ducts where hypoxic or anoxic conditions are present) [111].

## 5. Conclusions

Our current knowledge of chronic biofilm infections does not provide any simple solutions that allow us to cope with the observed increased antibiotic tolerance. Efficient antibiotics or treatment regimens which are able to completely clear all biofilms within a well-established chronic bacterial infection remain to be identified. In an effort to overcome the antibiotic tolerance in chronic bacterial infections, it is imperative that researchers and clinicians better understand and identify the chemical microenvironments that are characteristic of the biofilms encountered in chronic infections. Limitation of O_2_ seems a common feature of such microenvironments. This feature imposes a limitation on bacterial growth and forces cells to employ metabolic strategies other than aerobic respiration—for example, fermentation or denitrification. Anaerobic metabolism does not sustain as much growth and exploitation of electron donors as unrestricted aerobic respiration. These assumptions are supported by the reportedly slow bacterial growth and relatively high amounts of available nutrients—such as carbohydrates, proteins and fatty acids, which can be used as electron donors—in most studied chronic infections. Since chronic biofilm infections are as incredibly complex as they are, we can only touch the tip of the iceberg in explaining how biofilms persist in infections. However, there are various indications that metabolic activity is the core of antibiotic tolerance in biofilm infections.
